# Do We Need a New Diagnostic Model for Lung Cancer—Are We Ready? A Narrative Review of European Rapid Diagnostic Programs and an Operational Unified FTC-LCU Model

**DOI:** 10.3390/cancers18071167

**Published:** 2026-04-04

**Authors:** Joanna Maksymowicz-Jaroszuk, Lukasz Minarowski, Robert Marek Mroz

**Affiliations:** 1 2nd Department of Lung Diseases, Lung Cancer and Internal Diseases, University Teaching Hospital, 15-540 Bialystok, Poland; joanna.maksymowicz-jaroszuk@uskwb.pl (J.M.-J.); lukasz.minarowski@umb.edu.pl (L.M.); 2 Department of Respiratory Physiopathology, Medical University, 15-540 Bialystok, Poland; 3 2nd Department of Lung Diseases, Lung Cancer and Internal Diseases, Medical University, 15-540 Bialystok, Poland

**Keywords:** lung cancer, diagnostic delay, fast track clinic, rapid access clinic, lung cancer unit, coordinated care, turnaround time, multidisciplinary team, tumor board, Europe

## Abstract

Background: Lung cancer (LC) remains the leading cause of cancer-related mortality worldwide. Survival outcomes are strongly stage-dependent. Many patients are diagnosed at advanced stages due to pre-clinical and diagnostic delays. While advances in imaging, bronchoscopic techniques, molecular diagnostics, and systemic therapies have improved individualized treatment, system-level delays continue to limit their impact. Aim of the study: The aim of this narrative review is a synthesis with an implementation-oriented framework proposal. Part I synthesizes the peer-reviewed literature, Part II presents an operational framework integrating a Fast Trac Clinic (FTC) and a network of Lung Cancer Units (LCUs) including proposed turnaround-time (TAT) goals. Methods: A narrative review of the literature of selected European policy documents addressing diagnostic delays, rapid-access lung cancer pathways, and coordinated care models was conducted. Results: European models demonstrate that structured referral criteria, centralized coordination, and predefined interval targets can achieve the first specialist assessment within 7–10 days and the completion of diagnostics within 21–28 days in optimized settings. Key determinants of timeliness include: direct primary care referral, parallel diagnostic processes, prioritized pathology and molecular testing, and multidisciplinary team (MDT) assessment. We propose operational TAT targets for chest CT, PET-CT, histopathology, NGS, PFTs, and MDT decision-making. Conclusions: Reducing avoidable diagnostic and therapeutic delays in LC requires a coordinated, system-level approach. A standardized FTC-LCU pathway with explicit TAT benchmarks, multidisciplinary governance, and digital support infrastructure may improve diagnostic efficiency, increase the proportion of patients treated at earlier stages, and enhance patient experience. Prospective evaluation of implementation impact on stage distribution and survival is advised.

## Simple Summary

Lung cancer survival remains closely linked to stage at diagnosis, yet many patients are diagnosed at advanced stages due to fragmented and prolonged diagnostic pathways. Despite advances in bronchoscopic techniques, imaging technologies, molecular testing, and perioperative systemic therapies, only one in four Europeans is diagnosed at an early stage, and mortality remains high. Delays occur at multiple points, including before specialist referral, during diagnostic work-up, and prior to treatment initiation. This article reviews evidence on rapid-access lung cancer diagnostic models in Europe and proposes a coordinated Fast Track Clinic-Lung Cancer Unit (FTC-LCU) framework. We define measurable turnaround-time targets for key investigations and describe the governance structure and digital infrastructure required for implementation. By standardizing referral criteria, accelerating tissue acquisition and molecular testing, and auditing time intervals, coordinated pathways may increase early-stage diagnosis and improve patient experience, with potential survival benefits.

## 1. Introduction

Lung cancer (LC) is among the most common malignancies worldwide and remains the leading cause of cancer-related mortality in both men and women [1,2]. According to the latest WHO estimates, lung cancer accounted worldwide for 12.4% of all new cancer cases and 18.7% of all cancer deaths, and in Europe with 10.8% of incident cases and 18.9% of cancer deaths [3]. In Poland, approximately 22,000 new cases are diagnosed annually, corresponding to an incidence rate of approximately 57 per 100,000 population [4]. LC therefore constitutes one of the most significant oncological challenges for the Polish healthcare system [4]. Despite advances in imaging modalities, improvements in invasive diagnostic techniques, and the development of modern systemic therapies, overall survival in LC remains poor [5]. Prognosis continues to depend strongly on stage at diagnosis, with long-term survival largely limited to patients identified at early stages.

Across Europe and the United States, stage distribution at diagnosis is skewed toward advanced disease [5,6,7]. In many countries, the majority of patients present with stage III or IV disease, when curative-intent treatment options are restricted. In Poland, detailed nationwide stage-specific registry data remain limited. However, available estimates suggest that only 15–20% of cases are diagnosed at stages I–II. At these early stages, surgical resection combined with adjuvant or neoadjuvant therapy offers the greatest potential for cure. Approximately 20–25% of patients present at stage IIIA, a biologically and clinically heterogeneous category in which multimodal therapy remains feasible and may still provide meaningful long-term benefit. In contrast, the majority of patients are diagnosed at stages IIIB–IV, where disease is locally advanced or metastatic and treatment is predominantly systemic and palliative, focused on prolonging survival and preserving quality of life rather than achieving cure [8,9,10]. Earlier-stage diagnosis is closely associated with the availability, accessibility, and uptake of early detection programs, particularly low-dose computed tomography (LDCT) screening. Randomized controlled trials have demonstrated that LDCT screening reduces lung cancer mortality in high-risk populations, primarily by enabling the detection of tumors at earlier, potentially curable stages [11,12]. However, participation in organized screening programs remains limited in many European countries due to logistical barriers, limited awareness, socioeconomic disparities, and incomplete national implementation strategies. Moreover, comprehensive national data evaluating the long-term impact of screening programs on stage distribution and mortality are still scarce [13].

Beyond screening, reducing the time from first symptoms or from the detection of abnormal imaging findings to definitive diagnosis and treatment initiation represents a crucial system-level determinant of both clinical outcomes and patient experience [14]. Diagnostic and treatment intervals reflect the efficiency of referral pathways, imaging access, biopsy scheduling, pathology turnaround times, and coordination between care levels. In Europe, the interval from initial presentation to treatment initiation varies between approximately 12 and 16 weeks in individual countries, whereas worldwide, including in some South Asian countries, it may extend to around 21 weeks [15,16,17]. Such variability underscores the influence of healthcare organization and resource allocation on timeliness of care. Coordinated care has been defined by the World Health Organization as an approach integrating diagnostic, therapeutic, rehabilitative, and health promotion services across resources and settings [18]. In lung cancer, coordinated diagnostic pathways and integrated cancer networks have been associated with shorter time to diagnosis, earlier multidisciplinary discussion, and improved access to biomarker testing required for modern precision oncology [19,20]. However, shortening the diagnostic time in symptomatic patients alone does not necessarily translate into a higher proportion of early-stage diagnoses. Population-based analyses frequently demonstrate the so-called “waiting time paradox”, whereby patients with more aggressive or advanced disease often experience shorter diagnostic intervals due to more severe or rapidly progressive symptoms, while early-stage cases may have longer intervals because of subtle or nonspecific presentations [21,22]. This paradox complicates simple interpretations of time-to-diagnosis metrics and highlights the need for a nuanced evaluation of pathway performance. Accordingly, health services for patients with suspected lung cancer should be delivered within clearly defined procedures and time-bound frameworks that integrate rapid diagnostics with structured multidisciplinary decision-making [23]. Transparent interval definitions, measurable turnaround-time targets, and coordinated referral mechanisms are essential components of high-quality lung cancer care.

This article is structured as a dual-purpose contribution. First, it reviews diagnostic delay as a contributor to advanced-stage presentation and synthesizes evidence on European rapid-access models designed to streamline the diagnostic process (Part I: Section 3 and Section 4). Second, building on this evidence synthesis and on a retrospective workflow analysis conducted at the Białystok Lung Cancer Unit, it proposes a Fast Track Consultation-Lung Cancer Unit (FTC-LCU) operational framework applicable to the Polish healthcare system, emphasizing operational definitions of diagnostic intervals, explicit turnaround-time targets, and the coordination infrastructure required for effective nationwide implementation (Part II: Section 5, Section 6, Section 7, Section 8, Section 9 and Section 10). This hybrid design follows an established tradition in the health-services and clinical-pathway literature, whereby narrative evidence synthesis serves as the methodological foundation for context-specific framework development and policy recommendations.

## 2. Materials and Methods

This manuscript employs two complementary methodological approaches. The evidence synthesis component (Part I) is based on a narrative review of peer-reviewed literature, European national cancer policy documents, and publicly available institutional reports on rapid diagnostic lung cancer pathways.

The literature search was conducted in PubMed and Scopus covering publications from January 2010 to November 2025, including combinations of the following terms: “lung cancer”, “diagnostic delay”, “fast track”, “rapid access clinic”, “lung cancer unit”, “coordinated care”, “diagnostic pathway”, and “turnaround time”. European national cancer strategy documents and clinical guidelines were additionally retrieved through targeted searches of governmental and institutional websites. Title and abstract screening was performed independently by two authors (J.M-J. and L.M.), with discrepancies resolved by consensus [J.M-J, L.M., R.M.] Selection criteria prioritized original studies reporting pathway-level time intervals, institutional implementation models, national policy documents, and systematic reviews or meta-analyses addressing diagnostic timeliness in lung cancer. Given the narrative character of the manuscript, emphasis was placed on implementation-relevant publications and real-world pathway models rather than meta-analytic aggregation. As this is a narrative review, a PRISMA flow diagram and formal risk-of-bias assessment were not applied; however, the search strategy and selection rationale are reported in accordance with the recommendations of the SANRA (Scale for the Assessment of Narrative Review Articles) checklist [24].

The operational framework component (Part II) was additionally informed by a retrospective institutional workflow analysis conducted at the 2nd Department of Lung Diseases, Lung Cancer and Internal Diseases, University Teaching Hospital in Białystok (the Białystok Lung Cancer Unit). This analysis involved structured mapping of the diagnostic pathway for patients with suspected lung cancer evaluated between 2018 and 2025, including: (1) identification of all sequential and parallel diagnostic steps required for complete staging and treatment qualification; (2) documentation of observed turnaround times for each step under routine clinical conditions; (3) identification of rate-limiting steps and logistical bottlenecks; and (4) assessment of the minimal achievable pathway duration when scheduling was optimized through centralized coordination. This operational mapping was conducted as a quality-improvement exercise rather than a formal research study, and its findings are used to ground the proposed TAT targets in observed clinical practice rather than purely theoretical assumptions.

The conceptual framework is structured around three diagnostic intervals (preclinical, clinical diagnostic, therapeutic) and operational turnaround-time benchmarks derived from the combined evidence synthesis and institutional analysis.

## 3. Diagnostic Delay: Definitions and Clinical Consequences

We propose conceptualizing diagnostic delay as a structured, multi-interval process that may be perceived as comprising three principal components: (1) preclinical delay (from symptom onset or first healthcare contact to referral for specialist evaluation), (2) clinical diagnostic delay (from referral to histopathological confirmation and completion of staging), and (3) therapeutic delay (from diagnosis or multidisciplinary decision to treatment initiation) [25,26,27]. This three-interval framework allows for differentiation between patient-level, primary care-level, and system-level determinants of timeliness, and facilitates operational measurement of pathway performance.

Preclinical delay is frequently influenced by the non-specific nature of early lung cancer symptoms, including chronic cough, dyspnea, fatigue, chest discomfort, or weight loss. These symptoms may overlap with chronic respiratory conditions such as COPD or asthma and are often initially managed empirically in primary care. Repeated empirical treatment, delayed imaging referral, or under-recognition of risk in high-risk individuals may substantially prolong this interval [21,28,29]. Additionally, patient-related factors such as low symptom awareness, smoking-related stigma, socioeconomic disadvantage, and limited healthcare accessibility may contribute to prolonged time to specialist referral [30].

Clinical diagnostic delay reflects healthcare system capacity and coordination. Limited access to timely CT imaging, PET-CT, endobronchial ultrasound (EBUS), transthoracic biopsy, or interventional pulmonology services may create procedural bottlenecks. Furthermore, pathology turnaround times and access to comprehensive molecular testing—including EGFR, ALK, KRAS, BRAF, and PD-L1 assessment—may significantly extend the time to definitive staging and treatment planning [31,32,33]. Sequential rather than parallel diagnostic processes, fragmented communication between care levels, and limited integration of electronic referral systems further contribute to prolongation of this interval. Therapeutic delay encompasses the time between histopathological confirmation or multidisciplinary team (MDT) decision and the initiation of surgery, radiotherapy, chemotherapy, immunotherapy, or targeted therapy. Delays at this stage may arise from operating room capacity constraints, radiotherapy scheduling, reimbursement authorization, or pending molecular test results required for personalized treatment selection. Delays may have important clinical consequences, primarily through tumor progression, nodal involvement, or the development of distant metastases, potentially reducing eligibility for curative-intent surgery or combined-modality therapy. Although the causal association between delay and survival is complex and may be confounded by tumor aggressiveness, avoidable system-level delays remain clinically relevant. In addition to biological impact, prolonged diagnostic intervals have significant psychosocial consequences, including heightened anxiety, uncertainty, loss of trust in the healthcare system, and reduced patient-reported quality of life. Therefore, urgent pathway redesign is required. Such redesign should focus on reducing avoidable waiting times, enabling parallel diagnostic processes, maintaining diagnostic accuracy, and ensuring guideline-concordant, biomarker-informed treatment selection. Importantly, performance evaluation should rely on clearly defined interval metrics corresponding to the three-delay framework described above.

## 4. European Fast-Track and Rapid Access Clinic Models

Over the past 15 years, multiple European health systems have implemented rapid diagnostic pathways—commonly referred to as Fast Track Clinics (FTCs) or Rapid Access Clinics—for patients with suspected lung cancer [17,33]. These pathways typically enable direct referral from primary care to a specialist thoracic unit and coordinated progression through imaging, tissue acquisition, and multidisciplinary evaluation. Centralized scheduling, predefined timelines, and integrated care teams represent core components of these models. In the United Kingdom and Ireland, the reported models aim to provide first specialist assessment within 7–10 days of referral and completion of diagnostic workup within approximately 21–28 days. Treatment initiation is commonly targeted by day 42 in certain national programs, and these benchmarks are embedded in national cancer strategies as measurable quality indicators [17]. These systems emphasize transparency of interval metrics and routine performance monitoring. Similar initiatives have been described in Italy, including a “two-week wait” model to a dedicated pulmonary assessment point for suspected malignancy [34], as well as in Nordic countries, where integrated cancer care pathways combine standardized referral criteria, centralized coordination, and multidisciplinary evaluation [35]. These approaches aim to minimize fragmentation and reduce variability in access across regions. In contrast, healthcare systems in which primary care physicians cannot directly trigger rapid referral pathways face persistent access challenges, often resulting in prolonged diagnostic intervals and fragmented care trajectories [36]. To date, Poland has not implemented a nationwide lung cancer-specific fast-track diagnostic initiative comparable to those functioning in several Western and Northern European countries. This absence represents both a structural gap and an opportunity for system-level innovation.

## 5. Proposed FTC-LCU Unified Model

A nationwide rapid diagnostic system for suspected lung cancer may link Primary Health Care (PHC), Ambulatory Specialist Care (ASC), and reference centers organized as Lung Cancer Units (LCUs). The FTC component would provide a standardized entry point for patients with suspected malignancy, while the LCU network would ensure access to advanced imaging, tissue acquisition, pathology and molecular testing, multidisciplinary decision-making, and definitive treatment options.

**Figure 1:**
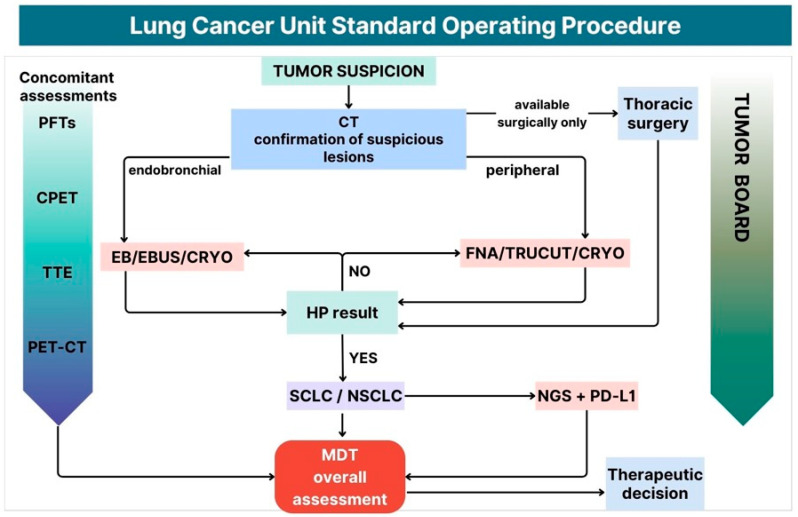
Lung Cancer Unit Standard Operating Procedure. The diagram illustrates the patient’s pathway from tumor suspicion through biopsies conducted to multidisciplinary decision-making and treatment initiation. Decision nodes include referral appropriateness assessment at the FTC entry point, tissue adequacy evaluation following initial biopsy, and branching for repeat procedures when initial tissue acquisition is non-diagnostic. The figure is intended as a conceptual operational schema; a fully detailed standard operating procedure including all exclusion criteria, contingency pathways, and clinical decision algorithms would be more appropriately presented in a separate institutional implementation manual. CPET—Cardiopulmonary Exercise Testing, CRYO—cryobiopsy, CT—Computed Tomography, EBUS—Endobronchial Ultrasound, FNA—Fine Needle Aspiration, HP—Histopathology Report, MDT—Multidisciplinary Team, NGS—Next-Generation Sequencing, NSCLC—Non-Small Cell Lung Cancer, PD-L1—Programmed Death-Ligand 1, PET-CT—Positron Emission Tomography-Computed Tomography, PFT—Pulmonary Function Test(s), SCLC—Small Cell Lung Cancer, TRUCUT—Core Needle Aspiration, TTE—Trans Thoracic Echocardiography.

A pragmatic model may begin with enhanced primary-care recognition of risk factors and alarm symptoms, structured referral criteria, and early radiologic verification. In high-risk patients or those presenting with suspicious symptoms, chest CT—preferably contrast-enhanced—should be readily accessible. Immediate routing of patients with suspicious findings into an FTC pathway would reduce fragmentation and avoid repeated intermediate consultations. Upon the identification of a suspicious lesion on CT ordered in primary care, the patient may be registered into a shared electronic coordination platform and routed to the nearest high-reference center for coordinated staging and tissue acquisition (Figure 1). As illustrated in Figure 1, the proposed model integrates rapid referral, centralized scheduling, parallel diagnostics (imaging, biopsy, pathology, and molecular testing), and early multidisciplinary discussion within predefined timeframes. Within the LCU, diagnostic procedures should proceed in a coordinated manner, ideally in parallel rather than sequentially. This includes staging imaging (contrast CT, PET-CT, brain MRI as indicated), tissue acquisition via bronchoscopy or percutaneous biopsy, rapid pathology reporting, reflex molecular testing, and prompt MDT review. Defined turnaround-time targets for each step would enhance accountability and system transparency. The unified FTC-LCU framework therefore combines standardized entry criteria, digital referral coordination, accredited high-reference centers, and measurable interval metrics. By linking primary care to specialized multidisciplinary units through clearly defined procedures and timelines (Figure 1), the model aims not only to reduce avoidable delays but also to standardize biomarker-informed treatment selection and improve overall pathway efficiency at a national level.

### 5.1. Primary Care Entry Criteria and Referral Safeguards

The effectiveness of a fast-track pathway depends critically on the appropriateness of referrals entering the system. To optimize the balance between sensitivity (ensuring that patients with genuine malignancy are rapidly evaluated) and specificity (avoiding pathway overload with low-probability cases), we propose the following structured referral criteria for direct FTC entry from primary care. Clinical indications warranting immediate FTC referral may include: (a) new or enlarging pulmonary nodule ≥ 8 mm identified on chest CT; (b) unexplained hemoptysis in a current or former smoker; (c) persistent respiratory symptoms (cough, dyspnea, chest pain) lasting more than 6 weeks in combination with at least one high-risk factor (age > 50, smoking history ≥ 20 pack-years, occupational exposure); (d) radiographic abnormality suggestive of malignancy (mass, mediastinal lymphadenopathy, pleural effusion) on chest radiograph or CT; (e) cytologically or histologically confirmed malignancy from any prior sampling procedure [37].

To mitigate the risk of excessive or inappropriate referral, several safeguards should be embedded in the pathway design. These include standardized electronic referral forms requiring the documentation of clinical findings and imaging results as a mandatory precondition for pathway activation; defined exclusion criteria to redirect clearly non-oncological presentations to alternative diagnostic tracks; and systematic feedback loops from the LCU to referring primary care physicians regarding the diagnostic outcome of each referral, enabling the continuous calibration of referral appropriateness. Such feedback mechanisms have been shown to improve referral quality in other European fast-track cancer pathways [38].

### 5.2. Distinguishing Features of the FTC-LCU Framework

While the proposed FTC-LCU model draws on established European rapid-access principles, several features distinguish it from existing fast-track systems. First, the framework integrates the FTC entry point and the LCU diagnostic-treatment infrastructure as a single, unified, nationally standardizable pathway with explicit accreditation criteria for participating centers. Existing models, such as the UK two-week-wait pathway or the Danish standardized cancer patient pathways, typically define time targets for individual intervals but do not prescribe a unified organizational architecture linking primary care referral, centralized diagnostic coordination, and multidisciplinary governance with defined coordinator roles (central coordinator, departmental coordinators, administrative dispatchers) within a single operational design.

Second, the FTC-LCU model introduces a dual-tier turnaround-time structure comprising a 10-day aspirational benchmark for optimized cases and a 28-day system-level standard for the broader population. This dual structure provides both an internal quality-improvement target and a realistic regulatory standard, whereas most existing models define a single target interval (e.g., 28 days to diagnosis, 62 days to first definitive treatment).

Third, the framework mandates the integration of reflex molecular testing (EGFR, ALK, ROS1, KRAS G12C, BRAF, PD-L1) as a mandatory component within the diagnostic TAT, rather than treating molecular characterization as a post-diagnostic add-on. This reflects the growing centrality of biomarker-informed treatment selection in modern lung cancer management and ensures that treatment-relevant molecular data are available at the time of the first MDT discussion.

Fourth, the model is specifically designed to address the structural gaps of the Polish healthcare system, including the absence of a national rapid-access lung cancer pathway, fragmented inter-level referral mechanisms, and limited digital coordination infrastructure. Rather than claiming that each individual component is entirely novel, we argue that the synthesis of these elements into a single, operationally defined, nationally applicable framework adapted to a system without pre-existing fast-track infrastructure constitutes the original contribution of this work.

## 6. Turnaround-Time Targets and Operational Requirements

Turnaround time (TAT) should be defined as the interval between ordering a diagnostic test and the availability of an actionable report in the medical record. This definition emphasizes clinical usability rather than mere technical completion of a procedure. An actionable report implies that results are formally validated, integrated into the electronic medical record, and available for multidisciplinary review and therapeutic decision-making. Based on procedural standards and institutional experience, minimal achievable TATs for key investigations can be set as operational targets while acknowledging that real-world performance will vary depending on system capacity, infrastructure, workforce availability, and patient complexity.

In the Bialystok Lung Cancer Unit (LCU), we analyzed the types of procedures required for complete staging and treatment qualification, their minimum procedural duration, and their clinically justified sequence. Importantly, the analysis considered both medical dependencies (e.g., the need for histopathological confirmation prior to molecular testing) and logistical dependencies (e.g., imaging scheduling, anesthesia availability, pathology workflow).

Our diagnostic model demonstrates that an overall 10-day diagnostic pathway represents a minimal timeframe achievable under optimal conditions. This benchmark is explicitly intended as an aspirational internal quality target and a proof-of-concept demonstrating what coordinated parallel diagnostics can achieve, not as a realistic standard applicable to all patients or all centers. Two critical conditions must be met: (1) continuous supervision by a central coordinator working closely with departmental coordinators and administrative dispatchers to pre-schedule diagnostic procedures in advance; and (2) a patient clinical profile that allows for the rapid completion of invasive diagnostics without delays related to clinical stabilization, comorbidity optimization, or management of acute complications.

For the broader patient population, we propose that an acceptable total time from first healthcare contact to completed diagnosis and MDT decision should not exceed 28 days. This system-level standard is aligned with established European benchmarks, including the NHS England 28-day Faster Diagnosis Standard and comparable Nordic pathway targets, and accommodates patients with comorbidities, those requiring repeat biopsies, or cases necessitating external molecular laboratory referral. Achievement of this target requires that administrative tasks are delegated to trained coordinators and supported by a centralized electronic coordination platform enabling real-time scheduling, monitoring, and escalation of delays.

**Figure 2:**
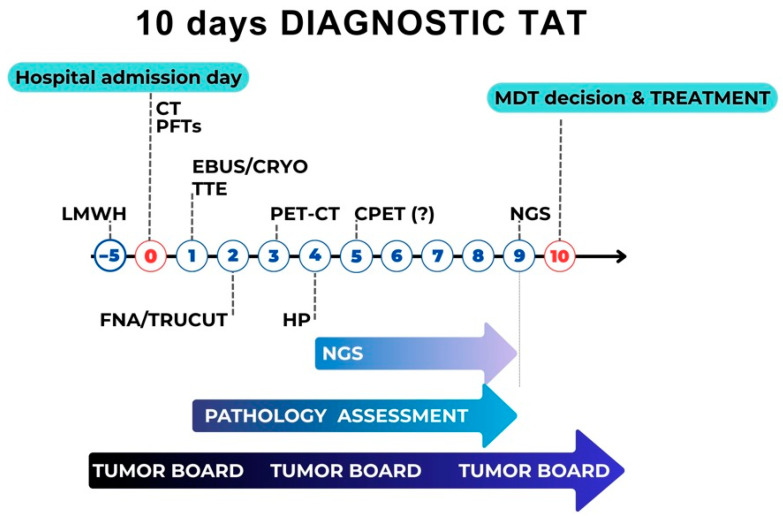
Proposed “10-day diagnostic TAT” protocol. The timeline represents the minimal achievable diagnostic pathway under optimal conditions (clinically stable patient, centralized coordination, dedicated diagnostic slots, in-house molecular platform). Annotations indicate: (a) conditions under which the 10-day aspirational benchmark applies versus when the 28-day system-level standard is triggered; (b) decision points for parallel versus sequential processing based on patient clinical status and tissue adequacy; (c) fallback routes for cases requiring repeat biopsy, liquid biopsy, or external molecular laboratory referral. This figure does not represent a universally applicable timeline; patient-specific factors and institutional capacity may necessitate pathway adaptation as discussed in the section on Patient Complexity and Pathway Adaptation. CPET—Cardiopulmonary Exercise Testing, CRYO—cryobiopsy, CT—Computed Tomography, EBUS—Endobronchial Ultrasound, FNA—Fine Needle Aspiration, HP—Histopathology Report, LMWH—Low-Molecular-Weight Heparin MDT—Multidisciplinary Team, NGS—Next-Generation Sequencing, PET-CT—Positron Emission Tomography-Computed Tomography, PFTs—Pulmonary Function Test(s), TRUCUT—Core Needle Aspiration, TTE—Trans Thoracic Echocardiography.

Proposed minimal TAT targets may include:

- Chest CT within 12 h from referral in high-suspicion cases;
- PET-CT within 4 days to examination plus 4 days for formal reporting;
- Histopathology within 3 days following tissue acquisition;
- Next-generation sequencing (NGS) within 5 days after histopathological confirmation;
- Pulmonary function testing (PFT) and cardiopulmonary exercise testing (CPET) within hours, where clinically indicated;
- Echocardiography within hours for surgical risk assessment (Figure 2).

Several of those targets require more precise context framing. The chest CT target of 12 h applies to inpatient or emergency presentations with high clinical suspicion and presupposes dedicated CT slots or 24-h radiology access. For outpatient referrals, a 48–72 h target represents a more realistic standard. The histopathology target of 3 days following tissue acquisition reflects rapid on-site assessment protocols and prioritized processing for suspected malignancy, as documented in several European pathology benchmarking initiatives [39,40]. The NGS target of 5 days is ambitious relative to current median turnaround times, which typically range from 10 to 14 days in many European laboratories; this target presupposes reflex testing protocols, in-house or closely affiliated molecular platforms, and dedicated bioinformatics capacity. Where external laboratory referral is required, a 10–14 day NGS TAT is acknowledged as the current realistic standard. The functional testing targets (“within hours”) refer to procedural duration and immediate availability in centers with dedicated pre-operative assessment clinics, and not to scheduling lead times in general outpatient settings.

When inpatient evaluation is required, these steps should be scheduled to occur in parallel whenever feasible, minimizing idle intervals between procedures. Central coordination is essential to ensure that imaging, biopsy, pathology, molecular testing, and pre-treatment functional assessment are synchronized within predefined timelines. This operational design reduces cumulative delay without compromising diagnostic accuracy or staging completeness.

Implementation of such TAT targets requires:

- dedicated scheduling slots for suspected lung cancer cases;
- pathology and molecular laboratories capable of prioritized processing;
- predefined reflex testing protocols to avoid administrative delay;
- an electronic dashboard tracking real-time interval performance;
- clearly defined escalation procedures when targets are exceeded.

The goal of establishing measurable TAT benchmarks is not merely acceleration, but standardization and predictability of care. By aligning procedural sequencing, coordination infrastructure, and accountability mechanisms, the proposed model seeks to transform diagnostic timelines from variable and reactive to structured and performance-monitored, as conceptually summarized in Figure 2.

### Patient Complexity and Pathway Adaptation

The parallel 10-day diagnostic model represents the default standard operational procedure (SOP) intended for patients with a good performance status (e.g., ECOG 0–1), admitted as scheduled appointment with tests arranged by the site coordinator. However, real clinical practice is diverse, so any pathway must account for patient heterogeneity. Common scenarios may require deviation from the accelerated parallel model, and the FTC-LCU framework should therefore include built-in flexibility.

First, in patients with insufficient tissue or non-diagnostic initial biopsy, repeat tissue acquisition may be required before histological diagnosis and molecular testing can proceed. In such cases, the 10-day minimal pathway will not be achievable, and the 28-day system-level target should accommodate at least one repeat procedure. Liquid biopsy may serve as a complementary approach when re-biopsy is not feasible or when rapid molecular characterization is needed to guide urgent treatment decisions.

Second, patients with significant comorbidities and border clinical status ECOG 2 (e.g., severe COPD with limited respiratory reserve, cardiovascular instability, anticoagulation requiring bridging protocols, or renal impairment precluding contrast-enhanced imaging) should be optimized before invasive diagnostic procedures can be safely performed. In these patients, a sequential rather than parallel diagnostic approach may be clinically appropriate, and the pathway timeline should be adapted accordingly without being considered a quality failure [41].

Third, patients presenting acutely with respiratory failure, massive hemoptysis, superior vena cava syndrome, or other oncological emergencies require immediate clinical management before elective diagnostic workup can commence. The FTC-LCU model primarily addresses the elective diagnostic pathway; emergency presentations necessitate distinct clinical protocols, and the formal pathway clock should commence only after clinical stabilization is achieved [42].

The model is designed to reflect real-world clinical complexity rather than an idealized “perfect patient” pathway. It defines a structured default framework while explicitly allowing for deviations driven by clinical factors. Monitoring should therefore include both completion rates within the 10- and 28-day targets and the documented reasons for deviations, enabling the separation of system-related delays from clinically justified extensions. This approach supports continuous pathway optimization by identifying modifiable inefficiencies without penalizing necessary clinical flexibility.

## 7. Coordinating Team and Multidisciplinary Governance

A dedicated coordinating team is essential for pathway reliability and continuity of care. The effectiveness of a fast-track diagnostic system depends not only on procedural standards, but also on clearly assigned responsibility and accountability. The coordinating structure could include a main coordinator responsible for overall pathway supervision, clinical coordinators representing pulmonology, oncology, radiology, pathology, thoracic surgery, and radiotherapy, and administrative staff such as dispatchers, medical secretaries, and registrars. The main coordinator may serve as the patient’s primary point of contact throughout the diagnostic and pre-treatment process. This role includes ensuring the completion of scheduled procedures within predefined turnaround-time targets, tracking progress across departments, resolving scheduling conflicts, and maintaining active communication among clinical stakeholders. In addition, the coordinator can provide patients with information regarding planned investigations, expected timelines, and next steps, thereby reducing uncertainty and improving patient experience. Weekly coordination meetings enable the systematic review of ongoing cases, identification of bottlenecks, and real-time adjustment of scheduling priorities. These meetings also provide an opportunity to monitor pathway performance against predefined targets and to initiate corrective measures when delays occur. Once histopathology and staging are available, each case may be discussed by a multidisciplinary team to ensure evidence-based, biomarker-informed treatment selection. MDT governance remains central to modern lung cancer care, as it integrates surgical, medical, radiotherapeutic, radiologic, and pathologic perspectives into a unified decision-making process.

## 8. Digital and AI Enablers

Information technology can substantially support coordinated pathway management at multiple levels. To provide an honest appraisal of the current state of digital enablement, we distinguished three tiers: currently implemented tools, proposed near-term components, and early-stage or speculative possibilities.

Currently implemented tools. Several digital tools relevant to FTC-LCU pathway support are already operational in clinical settings. In Poland, the “xLungs” is a newly developed algorithm for xAI assisted radiologic assessment and triage of chest imaging, assisting radiologists in the identification and prioritization of suspicious findings [42,43]. The implementation of ML/DL in oncological imaging has also been intensively studied and implemented [44,45,46] in lung cancer [47,48].

Institutional electronic scheduling and patient-tracking systems are in use at many European reference centers and provide the operational backbone for turnaround-time monitoring. National e-health platforms, such as Poland’s e-Zdrowie system, provide a potential infrastructure foundation for pathway-specific digital modules.

Proposed near-term components. Integrated electronic referral platforms linking primary care to LCUs would enable standardized referral documentation, automated pathway activation upon the submission of defined criteria, and real-time visibility of scheduling availability. Automated alerts for approaching turnaround-time thresholds—triggering escalation procedures when predefined TAT milestones are at risk—represent a technically feasible enhancement with clear operational value. Broader European initiatives illustrate the potential of organizational and digital innovation: mobile LDCT screening units such as the SOLACE program aim to increase participation among high-risk populations by reducing geographic and logistical barriers [49], while the OPTIMA initiative integrates real-world evidence and digital decision-support tools to optimize care pathways and resource allocation [42].

Early-stage or speculative possibilities. AI-assisted image interpretation for the automated detection and classification of pulmonary nodules on chest radiographs and CT scans [36,48] may further enhance early detection and triage, particularly in settings with limited radiology capacity; however, validation, regulatory approval, and integration into clinical workflows remain ongoing challenges. More advanced concepts, such as AI-driven predictive scheduling algorithms or natural language processing for automated extraction of clinical data from electronic records, are not yet validated for routine clinical use in this context and should be regarded as research priorities rather than near-term implementation components [50].

Digital infrastructure and AI support should not replace clinical expertise but rather augment coordination, transparency, and diagnostic precision within the FTC-LCU framework. Importantly, any digital tool deployed in this context must comply with applicable data protection regulations, including the European General Data Protection Regulation (GDPR) and national equivalents [51].

## 9. Monitoring, Evaluation, and Key Performance Indicators

We strongly recommend that the implementation of a rapid diagnostic pathway be accompanied by continuous audit using harmonized definitions of interval metrics. Without standardized measurement, comparison across centers and the evaluation of system performance remain unreliable.

Recommended key performance indicators (KPIs) include:

1. Time from first presentation to first chest imaging.
2. Time from abnormal imaging to specialist assessment.
3. Time to tissue acquisition.
4. Pathology and molecular testing turnaround times.
5. Time to MDT decision.
6. Time to treatment initiation.

These metrics correspond to the preclinical, clinical diagnostic, and therapeutic intervals defined earlier and allow for the identification of delay sources at specific pathway stages. Monitoring should include not only median values but also variability and outlier analysis. Patient-reported outcomes, including validated distress or anxiety measures, can complement operational metrics. Incorporating patient experience into evaluation frameworks ensures that pathway optimization addresses psychosocial as well as procedural dimensions of care. Transparent reporting of KPIs at institutional and national levels may foster accountability and continuous quality improvement.

## 10. Limitations and Implementation Considerations

Several limitations of this work should be acknowledged. First, as a narrative review, the literature selection process is inherently subject to potential selection bias. Although we have reported our search strategy, databases, search terms, and selection criteria, the absence of a systematic search protocol with formal risk-of-bias assessment means that some relevant publications may have been overlooked, and the evidence synthesis may disproportionately reflect pathways and models with published outcome data.

Second, the proposed FTC-LCU model is intended as a structured, practice-oriented framework rather than a definitive prescriptive standard. It has not yet been prospectively evaluated in a controlled implementation study. The institutional workflow analysis that informed the proposed TAT targets was conducted at a single center (the Białystok Lung Cancer Unit).

Third, the feasibility of proposed TAT targets will depend substantially on local and national healthcare capacity. Several specific barriers merit discussion.

Workforce constraints: Shortages of pulmonologists, thoracic surgeons, pathologists, and molecular biologists in Poland—particularly outside metropolitan academic centers represent a major barrier to nationwide implementation.

The proposed coordinator roles (central coordinator, departmental coordinators, administrative dispatchers) require dedicated funding, formal job descriptions, and institutional support that may not be readily available in all settings.

Inter-center inequality: Substantial heterogeneity exists across Polish pulmonology and oncology centers in terms of available equipment (e.g., EBUS, PET-CT scanners), pathology laboratory capacity, and molecular testing access. Implementation of the FTC-LCU model on a national scale would likely require a stratified, hub-and-spoke configuration linking smaller centers with limited diagnostic capacity to fully equipped reference LCUs.

Reimbursement and financing: The current reimbursement framework of the Polish National Health Fund (NFZ) does not include dedicated financing for rapid-access diagnostic pathways, pathway coordinator roles, or accelerated molecular testing. Legislative and financial reforms, including dedicated pathway reimbursement codes or bundled diagnostic tariffs, would likely be necessary to support sustainable implementation.

Pathology and molecular testing capacity: The proposed histopathology and NGS turnaround times are highly dependent on laboratory staffing, case volume, equipment availability, and whether molecular testing is performed in-house or referred to external laboratories. Achievable TATs will vary substantially across institutions, and the aspirational 10-day pathway may be realistic only at high-volume centers with integrated molecular platforms.

Digital interoperability: Integration of electronic referral, scheduling, and real-time tracking systems across primary care, ambulatory specialist care, and hospital-based LCUs presupposes a level of digital infrastructure and system interoperability that is not yet uniformly available across the Polish healthcare system. Polish national initiatives such as e-Health (e-Zdrowie), Individual Patients’ Account (Indywidualne Konto Pacjenta) and mCitizen (mObywatel) provide a foundation for further development and integration.

Patient-related factors, such as comorbidities, poor performance status, or the need for clinical stabilization prior to invasive procedures, may also influence achievable timelines in individual cases, as discussed in the section on Patient Complexity and Pathway Adaptation.

Finally, the manuscript does not include a formal cost-effectiveness analysis. While the economic rationale for pathway coordination is supported by evidence from other European systems, a detailed economic evaluation specific to the Polish context was beyond the scope of this narrative review and would require prospective implementation data. We identify this as a priority for future research. In Poland, system-level implementation will require legislative alignment of referral pathways, formal authorization of rapid-access mechanisms, accreditation criteria for LCUs with minimum volume thresholds, and harmonized reporting standards to ensure equity of access and consistent quality nationwide.

## 11. Future Directions

Future research should prospectively evaluate the impact of FTC-LCU implementation on stage distribution, diagnostic interval metrics, molecular testing completeness, treatment timeliness, patient-reported experience, and survival outcomes. Comparative effectiveness studies between regions with and without coordinated pathways—ideally employing stepped-wedge or quasi-experimental designs—would provide high-quality evidence supporting or refuting policy adoption.

A formal systematic review and meta-analysis comparing diagnostic pathway interventions across European healthcare systems, with quantitative pooling of time-interval and stage-distribution data where methodological heterogeneity permits, would complement the narrative approach of the present work and provide more robust evidence for policy decisions.

A dedicated cost-effectiveness analysis of the FTC-LCU model—incorporating direct pathway costs (coordinator salaries, digital infrastructure, accelerated molecular testing), indirect cost savings (avoided emergency presentations, reduced diagnostic duplication, earlier treatment initiation), and quality-adjusted life-year (QALY) estimates—should be considered an essential component of any prospective implementation evaluation.

Integration of FTC-LCU pathways with LDCT screening programs and AI-supported imaging triage warrants dedicated investigation. As screening programs expand across Europe, the interface between screening-detected abnormalities and rapid diagnostic pathways will become an increasingly important organizational challenge. Finally, multi-center validation of the proposed TAT targets across institutions with varying resource profiles would help identify which benchmarks are broadly achievable and which require adjustment based on local capacity.

## 12. Conclusions

Reducing avoidable delays in lung cancer diagnosis requires a coordinated pathway spanning primary care, specialist services, and a structured network of Lung Cancer Units. European rapid-access models demonstrate that timely specialist assessment and completion of diagnostics within predefined intervals are achievable when referral criteria, coordination mechanisms, and turnaround-time targets are explicit and routinely audited.

A standardized FTC-LCU framework supported by a dedicated coordinating team and robust digital infrastructure should therefore be considered a strategic priority for healthcare systems currently lacking rapid-access lung cancer pathways. The primary expected benefits of such a framework include improved pathway reliability, reduced psychosocial burden of diagnostic uncertainty, enhanced patient experience, and standardization of biomarker-informed treatment selection.

While the potential for favorable stage migration and associated survival improvement provides a compelling rationale for pathway acceleration, the causal relationship between shorter diagnostic intervals and improved population-level survival outcomes remains complex and is not yet definitively established. Prospective implementation studies with rigorous evaluation of stage distribution, time-to-treatment, and long-term survival outcomes are warranted to validate this hypothesis and guide evidence-based policy adoption.

## Data Availability

Not applicable.

## Abbreviations

AI	Artificial Intelligence
ALK	Anaplastic Lymphoma Kinase (receptor)
ASC	Ambulatory Specialist Care
BRAF	B-Raf Proto-Oncogene, Serine/Threonine Kinase
COPD	Chronic Obstructive Pulmonary Disease
CPET	Cardiopulmonary Exercise Testing
CRYO	Cryobiopsy
CT	Computed Tomography
EBUS	Endobronchial Ultrasound
ECOG	Eastern Cooperative Oncology Group (status)
EGFR	Epidermal Growth Factor Receptor
FNA	Fine Needle Aspiration
FTC	Fast-Track Clinic
GDPR	General Data Protection Regulation
HP	Histopathology Report
KPI	Key Performance Indicator
KRAS	KRAS proto-oncogene
LC	Lung Cancer
LCU	Lung Cancer Unit
LDCT	Low-Dose Computed Tomography
LMWH	Low-Molecular-Weight Heparin
MDT	Multidisciplinary Team
ML/DL	Machine Learning/Deep Learning
NFZ	Narodowy Fundusz Zdrowia—Polish National Health Fund
NGS	Next-Generation Sequencing
NHS	National Health Service
NSCLC	Non-Small Cell Lung Cancer
PHC	Primary Health Care
PRISMA	Preferred Reporting Items for Systematic Reviews and Meta-Analyses
QALY	Quality-Adjusted Life Year
SANRA	Scale for the Assessment of Narrative Review Articles
SCLC	Small Cell Lung Cancer
SOP	Standard Operating Procedure
TAT	Turnaround Time
TRUCUT	Core Needle Aspiration
TTE	Trans Thoracic Echocardiography
WHO	World Health Organization
xAI	Explainable Artificial Intelligence
